# Familiar unbalanced complex rearrangements involving 13 p-arm: description of two cases

**DOI:** 10.1186/s13039-018-0400-6

**Published:** 2018-09-06

**Authors:** Donatella Conconi, Nicoletta Villa, Serena Redaelli, Elena Sala, Francesca Crosti, Silva Maitz, Miriam Rigoldi, Rossella Parini, Leda Dalprà, Marialuisa Lavitrano, Gaia Roversi

**Affiliations:** 1 School of Medicine and Surgery, University of Milano-Bicocca, Monza, Italy; 2 Medical Genetics Laboratory, San Gerardo Hospital, Monza, Italy; 3 Pediatric Genetic Unit, Pediatric Department of Monza Brianza per il Bambino e la sua Mamma (MBBM) Foundation, San Gerardo Hospital, Monza, Italy; 4 Pediatric Department of Monza Brianza per il Bambino e la sua Mamma (MBBM) Foundation, San Gerardo Hospital, Monza, Italy

**Keywords:** Copy number variations, Chromosome 13 p arm, Unbalanced translocation, FISH, Array-CGH

## Abstract

**Background:**

Copy number variations (CNVs) are largely known today, but their position is rarely established by fluorescence in situ hybridization (FISH) or karyotype analysis.

**Case presentation:**

We described two families with copy number gain in which FISH analysis with the specific subtelomeric probe of chromosome 4q and 7q evidenced a third signal at band 13p11.2. Genomic study by array comparative genomic hybridization defined the triple dose segment. In the first case, the duplicate tract is free of known genes, in the second one it contained three expressed genes.

**Conclusions:**

The CNV localization on the short arm of an acrocentric chromosome could explain the lack of phenotypic effect, being known the regulatory role of heterochromatin in the position-effect silencing. Furthermore, we would like to underline the importance of using complementary techniques such as FISH and array-CGH to obtain a better definition of genomic rearrangements.

## Background

Copy number variations (CNVs) are well studied and largely known today [1–3]. They may be losses or gains, extremely variable in size, which may or may not affect the phenotypes. For this reason, CNVs could be classified in pathogenetic, benign and with uncertain significance, as defined by online databases (for example http://dgv.tcag.ca/dgv/app/home, https://decipher.sanger.ac.uk/, or http://gvarianti.homelinux.net/gvariantib37/index.php).

Nowakowska et al. [4] described how some CNVs considered as de novo, are de facto inherited by parents carrying a cryptic translocation (not detectable with array comparative genomic hybridization technique). In these cases the unbalanced genomic tract, inherited in the affected proband, is not found in its canonical chromosomal position, but is translocated to another chromosome.

Instability of the short arm of acrocentric chromosomes (13, 14, 15, 21, 22) is well known, in fact qter satellite Y chromosome has considered a normal variant since 1995 [5]. Non-acrocentric chromosomes with satellites instead of telomeres in both p arm and q arm have also been described [6]. Furthermore, most of the small supernumerary chromosome markers derives from acrocentric chromosomes [7, 8]. Finally, even Robertsonian translocations, which have an incidence of about 1/1000 newborns, are the results of a rearrangement between acrocentric p arms [9].

We had the opportunity to study two non-traditional rearrangements that gave rise to partial trisomies without an apparent phenotypic effect. The first case showed a translocation involving chromosomes 4qter and 13p and the second involving chromosomes 7qter and 13p. In both cases, conventional cytogenetics analysis showed normal chromosomes, the fluorescence in situ hybridization (FISH) study with specific subtelomeric probes evidenced a third signal on chromosome 13p and finally genomic study by array comparative genomic hybridization (CGH) defined the triple dose segment.

## Cases presentation

### Materials and methods

#### Cytogenetic and FISH analysis

Peripheral blood metaphases were obtained from phytohaemagglutinin-stimulated lymphocytes, cultured with Synchro kit (Celbio) according to manufacturer’s protocol. Chromosome analysis was carried out applying QFQ banding according to routine procedures, and karyotypes were reconstructed following the guidelines of ISCN 2016 [10].

FISH analysis was carried out according to the manufacturer’s protocol for specific subtelomeric probes (Kit Chromoprobe Multiprobe-T System, Cytocell).

#### Array-CGH

Genomic copy number analysis was performed with array-CGH using the SurePrint G3 Human Genome CGH + SNP Micro-array Kit, 4 × 180 K (Agilent Technologies) following the manufacturer’s recommendations. The target DNA was extracted from the peripheral blood by Wizard Genomic DNA purification kit (Promega Corporation). DNA control reference was provided by Agilent (Agilent Technologies). The arrays were scanned at 3-μm resolution using an Agilent microarray scanner and analyzed using CyoGenomics 3.0 software (Agilent Technologies). The aberration detection method 2 (ADM-2) algorithm was used to compute and assist in the identification of aberrations for a given sample.

Significant chromosomal aberrations were determined using the algorithm ADM-2 with a threshold of 5 and a minimum absolute average log2 ratio of 0.25. Putative chromosome copy number changes were defined by intervals of 3 or more adjacent probes and were considered as being duplicated or deleted when results exceeded 0.25. All nucleotide positions were based on the Human Reference Sequence Assembly, February 2009 GRCh37/hg19 of the UCSC Genome Browser (http://genome.ucsc.edu/).

### Cases description

#### Case 1

The proband was the first child of a non consanguineous couple. He was born at 31 + 6 weeks of gestation by caesarean section due to maternal preeclampsia. At birth, a respiratory distress occurred and a feeding by gavage for three weeks was introduced. He showed growth difficulties and psychomotor delay. At 17 months prominent subcutaneous nodules next to hand joints, ankles, knees, spine were present, associated with joint swelling, neurological deterioration, cachexia due to feeding problems and mitralic regurgitation. The baby died at 23 months of age.

Farber disease was hypothesized and successively confirmed by undetectable levels of acid ceramidase (Laboratory prof. Thierry Levade, Toulose). Molecular analysis of N-acylsphingosine amidohydrolase (*ASAH1*) gene (8p22) showed the splicing mutation c.648 + 1G > C in intron 8 and the missense mutation c.1085C > G (p.Pro362Arg) in exon 13. The phenotype was consistent with the molecular findings. Parents were both carriers for this autosomal recessive disorder.

**Fig. 1:**
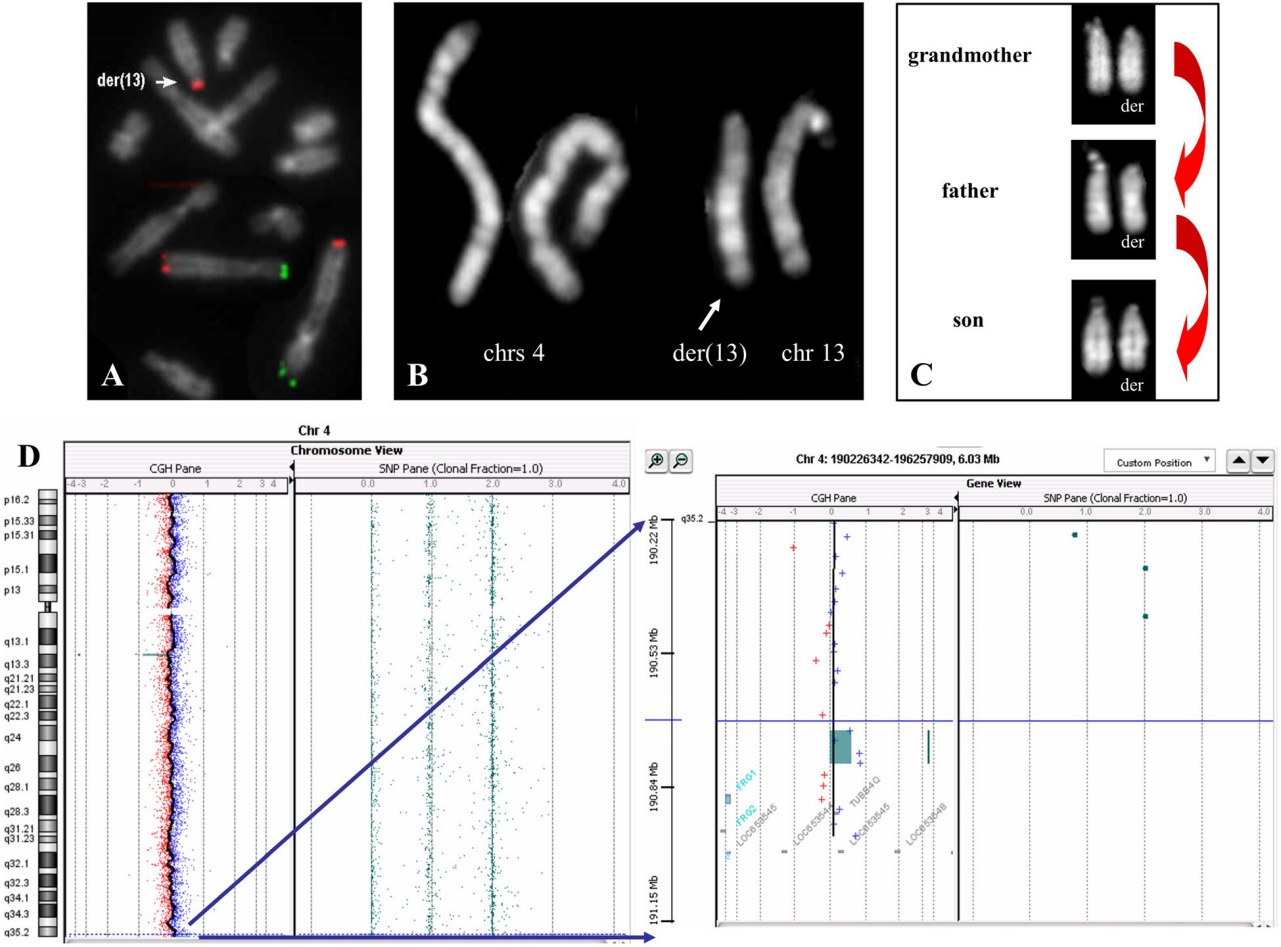
Genetic characterization of the first case. **a** FISH analysis with chromosome 4 specific subtelomeric probes: 4pter (green signals) and 4qter (red signals). **b** Proband’s QFQ-banded chromosomes 4 and 13 (700-band levels). **c** Normal (left) and derivative (right) chromosomes 13 in the proband, his father and grandmother. **d** Array-CGH view of chromosome 4 and enlargement of the duplicated region

At 12 months old, conventional karyotype analysis on peripheral blood evidenced an apparently normal karyotype The analysis of subtelomeric regions by means of FISH revealed the presence of a partial trisomy of the terminal region of the chromosome 4q located at 13p, resulting in a derivative chromosome 13. The ISCN description was: ish der(13)t(4;13)(q35;p11.2)(q35+)(DJ963k6+) (Fig. 1a, b). The father and the paternal grandmother were carriers of the same unbalanced translocation (Fig. 1c), as determined by FISH analyses (data not shown).

**Fig. 2:**
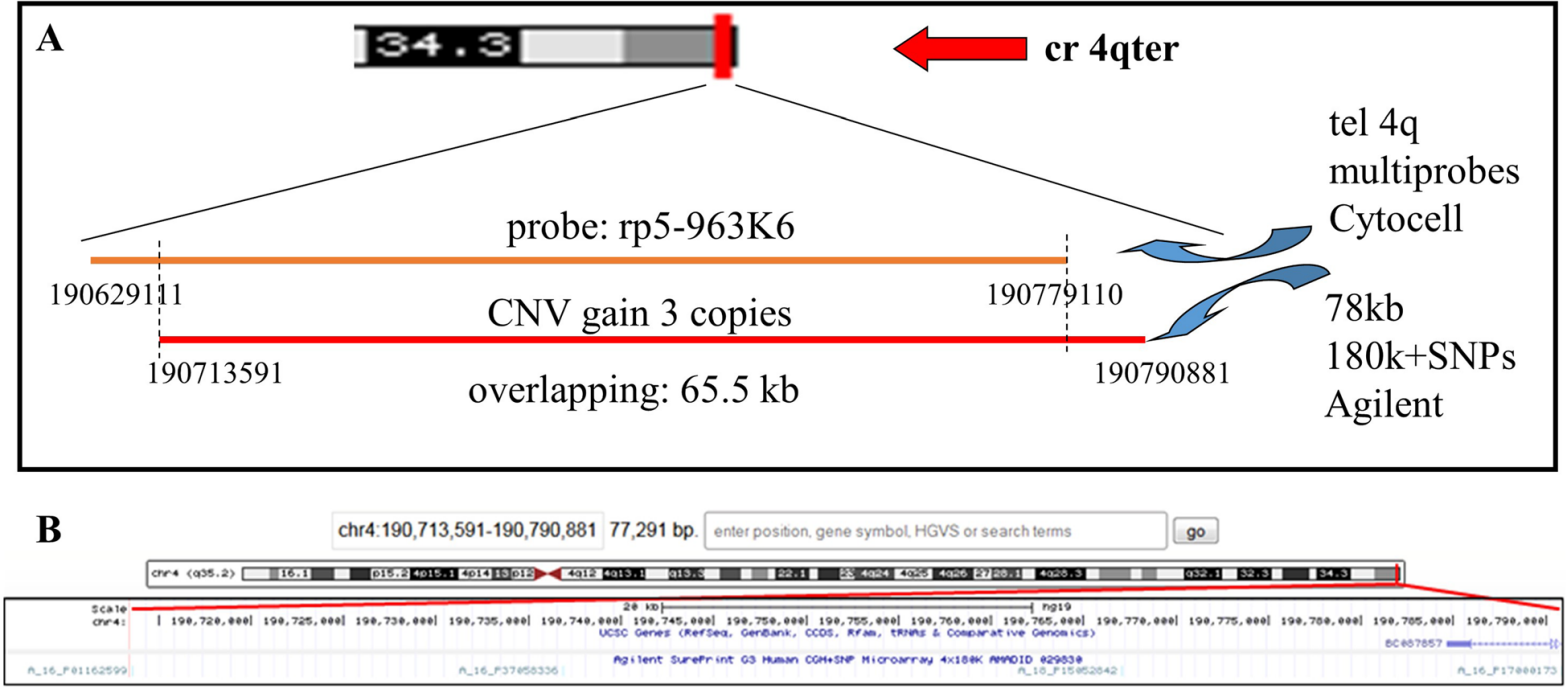
First CNV description. **a** 4q subtelomeric FISH probe sequence and CNV position. **b** UCSC region of the trisomic segment

In order to understand if 4q trisomy could be considered a polymorphism, an array-CGH analysis was performed in the father (Fig. 1d). Array-CGH analysis confirmed the gain at 4q, consisting in 4 probes partially overlapped the FISH probe. The trisomic region, of about 78 kb, is included within the *FRG1*-divergent transcript, a long non coding RNA (Fig. 2a, b). The 180 K array slide covers an additional region of other 6 disomic probes (about 142 kb), the last of which maps about 195 kb apart from the 4qter (region uncovered by array-CGH probes).

#### Case 2

This proband was investigated as the father of a single child affected by psychomotor delay and moderate facial dismorphisms, in whom subtelomeric FISH analysis identified a de novo loss of 9q.

**Fig. 3:**
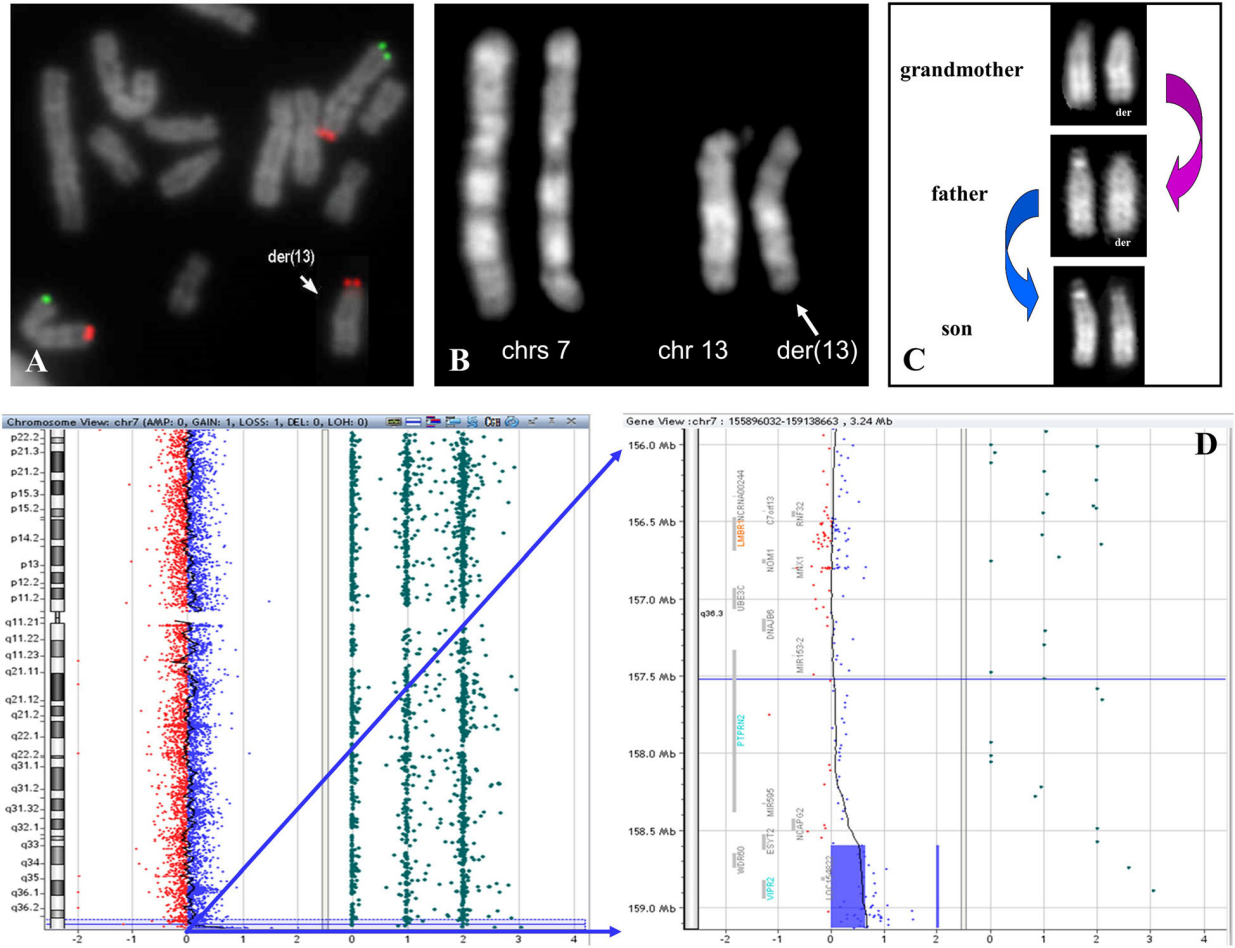
Genetic characterization of the second case. **a** FISH analysis with chromosome 7 specific subtelomeric probes: 7pter (green signals) and 7qter (red signals). **b** Father’s QFQ-banded chromosomes 7 and 13 (550-band levels). **c** Chromosomes 13 transmission in the child, his father and his grandmother. D) Array-CGH view of chromosome 7 and enlargement of the duplicated region

The same investigation in the proband showed an additional signal for the specific subtelomeric 7q probe on chromosome 13p, giving rise to a derivative chromosome 13 with a segmental 7q trisomy. The ISCN description was: ish der(13)t(7;13)(q36;p11.2)(q36+)(2000a5+) (Fig. 3a, b). FISH analyses allowed us to define the maternal inheritance of the rearrangement (data not shown), that was not transmitted to the affected child (Fig. 3c). The familial history was uneventful, neither consanguinity nor genetic diseases were reported.

**Fig. 4:**
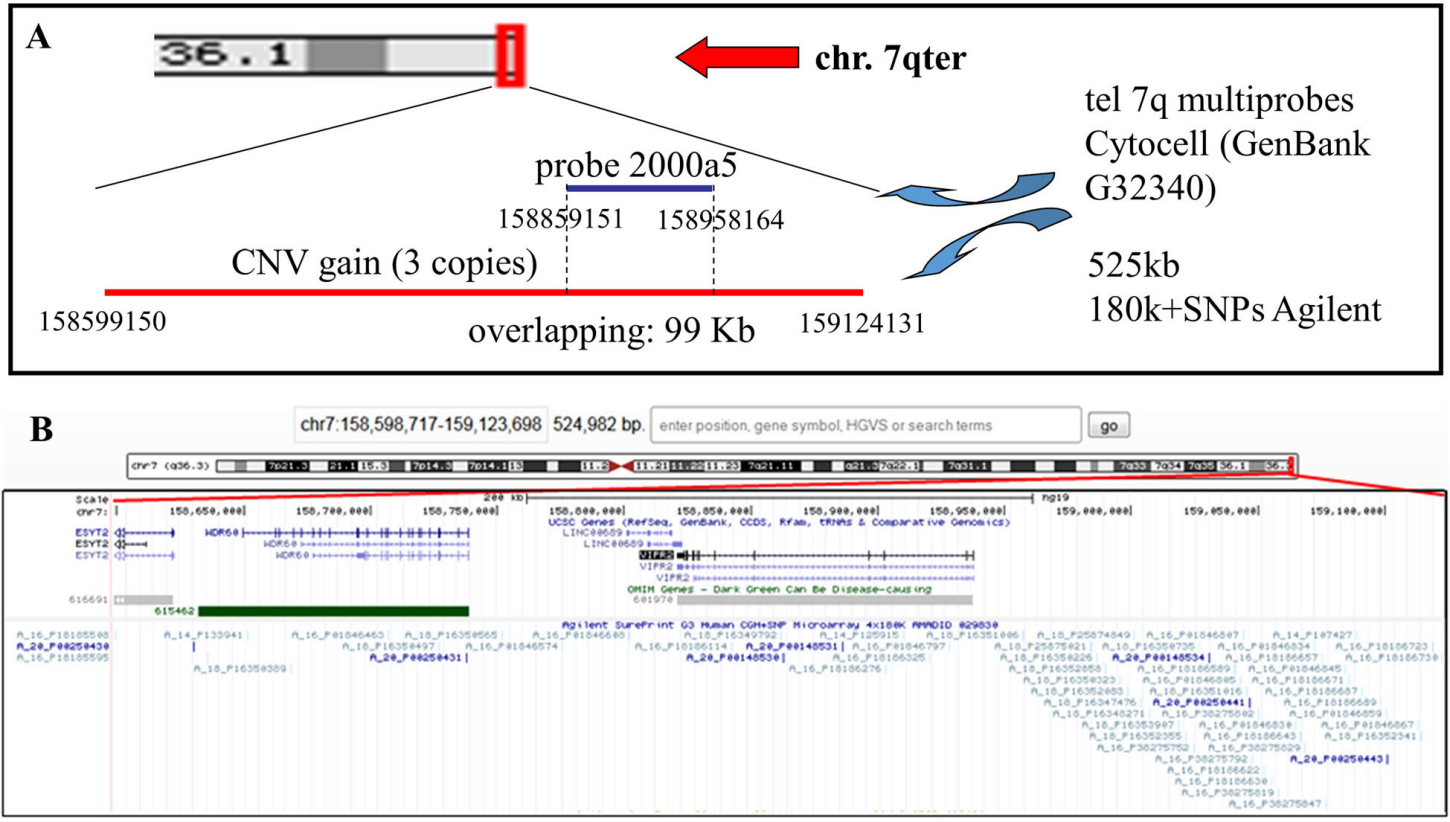
Second CNV description. **a** 7q subtelomeric FISH probe sequence and CNV position. **b** UCSC and OMIM genes in the trisomic region

In order to better characterize a trisomic segment, array-CGH analysis was performed in the proband, confirming a duplication of about 525 Kb at 7q36.3 (from nt 158,599,150 to nt 159,124,131) (Fig. 3d). This region is covered by 54 oligo and 7 SNPs probes with a copy number of 3 and includes the 7q telomeric specific FISH probe (from nt 158,859,151 to nt 158,958,164) (Fig. 4a). The exon 1 of *ESYT2* (OMIM616691) and the whole *WDR60* (OMIM615462) and *VIPR2* (OMIM601970) genes map within the region (Fig. 4b).

## Discussion and conclusions

Several studies about CNVs have been reported, but in very few cases chromosomes were performed in order to verify their position. Here we described two families with a copy number variation in which the gained segment is translocated to the 13p11.2 region that loses satellite sequences. This type of translocation could be underestimated because cytogenetically unrecognizable, in contrast to the cases in which a derivative chromosome acquires satellite sequences at the end of short or long arm (ps or qs chromosome).

The recurrence of cases with rearrangements involving acrocentric p arms and terminal region of other chromosomes suggests a predisposing mechanism. Intriguingly, Cazaux et al. [11] reported that the proximity between telomeres, centromeres and rDNA clusters of acrocentric chromosomes during meioses might facilitate their sequence homogenization by non-homologous recombination, according to the model of chromosome pairing proposed by Scherthan et al. [12].

In the first case the duplicate segment does not contain known genes to date, but is included within the *FRG1*-divergent transcript, a long non coding RNA. This CNV is reported, with higher size, in Troina Database and in the Database of Genomic Variants (DGV) as benign.

In the second case, the CNV is indicated as polymorphic variant in 0.04% of cases of DGV gold standard variant; Troina Database shows four overlapping CNVs all classified as benign and finally Decipher reports four CNVs, one inherited with unknown classification, one benign inherited, one of uncertain significance and the last inherited with autistic phenotype (partial uncertain significance). The trisomic segment contains three expressed genes: *WDR60*, associated to short-rib thoracic dysplasia 8 with or without polydactyly, *ESYT2,* that encodes for extended synaptotagmin-like protein 2 and is not associated to pathology and *VIPR2,* that has been reported in association with schizophrenia [13].

The copy number gain localization on the short arm of an acrocentric chromosome in this case could explain the lack of phenotypic effect, being known the regulatory role of heterochromatin in the position-effect silencing.

We would like to stress the importance of using complementary techniques such as FISH and array-CGH to obtain a better definition of genomic rearrangements.

## Abbreviations

CGH: Comparative Genomic Hybridization
CNV: Copy Number Variation
DGV: Database of genomic variants
FISH: Fluorescence In Situ Hybridization
